# Experience-dependent reshaping of body gender perception

**DOI:** 10.1007/s00426-021-01569-4

**Published:** 2021-08-13

**Authors:** Giulia D’Argenio, Alessandra Finisguerra, Cosimo Urgesi

**Affiliations:** 1 Department of Life Sciences, University of Trieste, Trieste, Italy; 2 Laboratory of Cognitive Neuroscience, Department of Languages and Literatures, Communication, Education and Society, University of Udine, via Margreth, 3, 33100 Udine, Italy; 3 Scientific Institute, IRCCS E. Medea, Pasian di Prato, Udine, Italy

## Abstract

Protracted exposure to specific stimuli causes biased visual aftereffects at both low- and high-level dimensions of a stimulus. Recently, it has been proposed that alterations of these aftereffects could play a role in body misperceptions. However, since previous studies have mainly addressed manipulations of body size, the relative contribution of low-level retinotopic and/or high-level object-based mechanisms is yet to be understood. In three experiments, we investigated visual aftereffects for body-gender perception, testing for the tuning of visual aftereffects across different characters and orientation. We found that exposure to a distinctively female (or male) body makes androgynous bodies appear as more masculine (or feminine) and that these aftereffects were not specific for the individual characteristics of the adapting body (Exp.1). Furthermore, exposure to only upright bodies (Exp.2) biased the perception of upright, but not of inverted bodies, while exposure to both upright and inverted bodies (Exp.3) biased perception for both. Finally, participants’ sensitivity to body aftereffects was lower in individuals with greater communication deficits and deeper internalization of a male gender role. Overall, our data reveals the orientation-, but not identity-tuning of body-gender aftereffects and points to the association between alterations of the malleability of body gender perception and social deficits.

## Introduction

Starting from the description of the Waterfall illusion, for which looking at a cascade and suddenly directing the eyes on the rocks result in the illusory perception of the rocky surface moving upwards (Addams, 1834), many psychophysical studies have targeted at visual aftereffects. This perceptive experience occurs after the prolonged exposure to an adaptor stimulus, which biases the perception of the following ones toward the opposite pole of the adaptor. Over the years, what observed for motion (Anstis et al., 1998) has been proven for many other basic properties of visual stimuli, like orientation (Gibson & Radner, 1937; He & MacLeod, 2001) and colours (Dodwell & Humphrey, 1990). Research arising from early paradigms mainly conceived aftereffects as retinotopic, since they appeared to be confined to the field of the retina that was exposed to the adaptor (Knapen et al., 2009). However, evidence that similar aftereffects can be present even when adaptor and test stimuli appear in different retinal regions have supported a spatiotopic explanation (Turi & Burr, 2012), especially for more complex stimulus dimensions. Across different levels of stimulus processing, aftereffects reflect a recalibration of the perceptual system according to a changing surrounding (Thompson & Burr, 2009), likely due to firing adaptation in populations of neurons coding for a specific stimulus feature (Barlow & Hill, 1963).

Many studies have demonstrated face aftereffects along various semantic and social continua (Webster et al., 2004), such as identity (Clifford & Rhodes, 2005; Leopold et al., 2001), expression (Butler et al., 2008; Ying & Xu, 2017), ethnicity (Webster et al., 2004) and gender (Kloth et al., 2010; Webster et al., 2004). These effects occur across changes in size and location (Rhodes et al., 2003; Yamashita et al., 2005; Zhao & Chubb, 2001), suggesting the involvement of high-level coding mechanisms. Nevertheless, evidence of reduced aftereffects when the adapting and testing faces differ for ethnicity (Gwinn & Brooks, 2013, 2015; Jaquet & Rhodes, 2008), gender (Little et al., 2005), and age (Yang et al., 2011) suggests that face aftereffects involve neural populations specifically tuned to these aspects of facial identity. Similarly, greater face-gender aftereffects occur when the orientation of the adapting and testing faces is congruent compared to when the faces are in opposing orientations, thus pointing to a partial orientation-tuning of the neural populations encoding face gender (Rhodes et al., 2004; Watson & Clifford, 2006).

More recently, aftereffects for human bodies, mainly related to body size and body shape, have been demonstrated (Brooks et al., 2019, 2020). As for faces, also these body aftereffects likely reflect high-level coding, as they occur in free-viewing conditions, where the testing and adapting stimuli occupy different retinal positions (Brooks et al., 2019). Furthermore, they transfer, albeit with different magnitude, across different orientations (Brooks et al., 2018), identities (Brooks et al., 2016; Hummel et al., 2012), genders (Brooks et al., 2019) and ethnicities (Gould-Fensom et al., 2019) of the adapting and testing stimuli. Therefore, visual adaptation has been proposed as one of the mechanisms that may be involved in the internalization of body-ideals in the general population (Burke et al., 2010; Challinor et al., 2017; Robinson & Kirkham, 2014) and the development of body misperception and disordered eating (Brooks et al., 2019, 2020; Griffiths et al., 2018). Indeed, adaptation to idealized body images presented by media may shift our point of subjective normality by, consequently, influencing the way we actually perceive our own and others’ bodies (Bould et al., 2018; Brooks et al., 2016; Hummel et al., 2012; Stephen et al., 2018; Mele et al. 2013; Winkler & Rhodes, 2005). Accordingly, an altered adaptation-related reshaping of body perception has been reported in patients with Eating Disorders (EDs; Mele et al., 2016; Mohr et al., 2016; Cazzato et al. 2016).

Other researchers have lingered on other properties of the human body that can be sensitive to visual adaptation, such as gender. Compared to shape and size dimensions, which are susceptible to recurrent changes by their nature, gender can be considered a naturally stable feature of a body. Furthermore_,_ a rapid processing of conspecifics’ gender plays a significant role in social interactions, being at the root of mate selection and reproductive behaviour (Singh & Singh, 2011; Windhager et al., 2011). Despite this inherent need for accurate discrimination and its stability, gender perception has been shown to be susceptible to aftereffects. Using silhouettes of headless human models, Palumbo and colleagues (2013) demonstrated that prolonged exposure to a distinctively male or female body leads to gender-specific aftereffects on the perception of an androgynous one. Interestingly, stronger effects were found, for either male or female participants, when they were adapted to silhouettes of their own sex, thus pointing to a high-level modulation of body aftereffects according to the meaning of the stimulus for the observer. That body-gender aftereffects are related to high-level stimulus dimensions has been also corroborated by findings of cross-categorical (i.e., body-to-face) aftereffects, where exposure to faceless body biases the perception of the gender and identity of faces (Ghuman et. al, 2010a). The opposite direction of cross-category aftereffects (i.e., face-to-body) has been also demonstrated (Palumbo et al., 2015). Cross-categorical gender aftereffects for faces and bodies point to a common, high-level representation of an individual’s gender conveyed by facial and bodily cues. Nevertheless, different cross-category studies, such as those testing the influence of gender-specific voices (Kloth et al., 2010) or hands (Kovács et al., 2006) on face perception, have failed to find adaptation effects. This suggests a closer relationship between body and face representations as compared to other gender-specific stimuli.

The perception of both faces and bodies seems to involve, more than other objects, configural processing, where a stimulus is encoded on the basis of the relations among its parts in the context of the whole stimulus space, and not only feature-based mechanisms, where the stimulus is encoded on the basis of the details of its single parts (Bartlett & Searcy, 1993; Murray et al., 2003; Bartlett & Searcy, 1993; Carey 1992; Freire et al. 2000; Leder & Bruce 2000). Indeed, different faces and bodies share a common template configuration that is shaped by repeated exposure to various exemplars of these two categories. This template representation is used to perceive individual face and body stimuli, at least when they are presented in a canonical, upright orientation, which is compatible with those stimuli that have contributed to the formation of the category template. Consequently, a configural processing mechanism may be used only for upright stimuli, explaining the disproportioned, as compared to other objects, drop of perceptual performance when faces and bodies are presented upside-down, with respect to the canonical upright orientation, the so-called inversion effect (Maurer et al., 2002; Reed et al., 2003). In this framework, aftereffects for upright and inverted faces have been attributed to specific adaptation of configural and local processing mechanisms, respectively (Watson et al. 2003; Webster & MacLin, 1999; Watson & Clifford, 2006; Rhodes et al., 2004). Interestingly, abnormal aftereffects for (upright) faces have been reported in children with autism (Pellicano et al., 2007), who have deficits in configural processing (Behrmann et al., 2006; Rondan & Deruelle, 2007), in their relatives (Fiorentini et al., 2012), and in typical developing men with high autistic traits related to social skills (Rhodes et al., 2013b).

In keeping with the notion that both configural and local processing mechanisms may be involved in the processing of upright faces, while only the latter may be involved for inverted faces, adaptation to upright faces has been shown to readily transfer to the perception of inverted faces, while the opposite transfer of effects is negligible (Watson & Clifford, 2006). Furthermore, the configural and local face-coding mechanisms can be contingently adapted since opposite size and gender aftereffects were induced simultaneously for upright and inverted faces by adapting to opposite distortions in each orientation, an evidence of contingent aftereffects (Rhodes et al., 2004). It is unclear, however, whether the same orientation-specificity of gender aftereffects also occurs for human bodies. A recent study showed that upright and inverted bodies have comparable aftereffects on the gender perception of upright and inverted faces, despite larger aftereffects were observed for the latter stimuli (Kessler et al., 2013). This finding suggests that bodies adapt an orientation-independent representation of faces, likely mediated by local processing mechanisms, involved for both upright and inverted faces processing.

In this study, we aimed to investigate the identity- and orientation-tuning of body-gender aftereffects, whilst evaluating the potential modulation of personality traits associated to body misperceptions and autistic traits. In particular, in three distinct experiments, we used a body adaptation paradigm, in which participants were exposed to a series of distinctively male or female adapting bodies and, then, they were asked to categorize the gender of androgynous testing bodies. To control for low-level stimulus features, renderings of computer avatars were manipulated to appear more or less feminine (D’Argenio et al., 2020), thus leaving intact low-level visual features, like color, luminance and size. In “Experiment 1”, we manipulated the individual body characteristics of the adapting and testing bodies to test whether body-gender aftereffects are identity-specific or transfer across different characters. Body identity here refers to those individual body characteristics (e.g., physique, skin texture and color, etc.) that characterize individuals independently from their gender. In Experiments 2 and 3, we also manipulated the orientation of the adapting and testing bodies, asking participants to categorize the gender of both upright and inverted androgynous bodies after adaptation to upright (Experiment 2) or upright and inverted (Experiment 3) gender-typical bodies. Generally, we expected that adaptation to male or female bodies would produce aftereffects in the perception of androgynous bodies, making them appear more feminine after male exposure and more masculine after female exposure. Evidence of identity tuning and orientation independence of body-gender aftereffects would call for high-level, object-based mechanisms involved in the processing of individual body characteristics. Conversely, their identity independence and orientation tuning would call for lower-level mechanisms involved in the processing of body form cues. Concerning the influence of specific individual traits, we expected a modulating role of autistic traits (Pellicano & Burr, 2012), body misperceptions (Bould et al., 2018; Brooks et al., 2016; Stephen et al., 2018; Mele et al. 2013; Mohr et al., 2016) and gender (Palumbo et al., 2013).

## Experiment 1

We tested whether body-gender aftereffects are specific for the individual characteristics of the adapting body or generalize across different characters. To this aim, we manipulated the gender distinctiveness of two different adapting avatars to appear highly (90%) or lowly (60%) masculine/feminine. The masculine or feminine versions of both characters were presented as adapting bodies during a body-pose categorization (i.e., static vs. dynamic) task, while the androgynous versions of the same characters were later presented as testing bodies in a body-gender (male vs. female) categorization task. If body-gender aftereffects involve the adaptation of populations of neurons tuned to the specific characteristics defining a body, we expected higher aftereffects for the high- than for the low-adapted characters. Conversely, if body-gender aftereffects involve the adaptation of populations of neurons encoding body forms independently from individual characteristics (Kable & Chatterjee, 2006; Wiggett & Downing, 2011), we expected comparable aftereffects for the two characters. Furthermore, in keeping with the finding that body-gender aftereffects are stronger when the adapting stimulus is of the same gender of the observer (Palumbo et al., 2013), we expected greater aftereffects in male and female participants after male and female adaptation, respectively.

### Materials and methods

#### Participants

A sample of 30 students (15 female, mean age 23.22, SD 4.54 years) from the University of Udine participated in the experiment in return for course credits. They reported normal or corrected vision and had no current neurological or psychiatric disorders as assessed with the Symptom Checklist 90 (SCL-90; Derogatis et al., 1973). Written informed consent was obtained from all participants. The procedures were approved by the local ethics committee (*Commissione di garanzia per il rispetto dei principi etici nell’attività di ricerca sugli esseri umani*, Department of Languages and Literatures, Communication, Education and Society, University of Udine; Prot. N. CGPER-2019-12-09-04) and conformed with the Helsinki Declaration. Participants were right-handed as ascertained with standard handedness inventory (Oldfield, 1971).

#### Stimuli

**Fig. 1:**
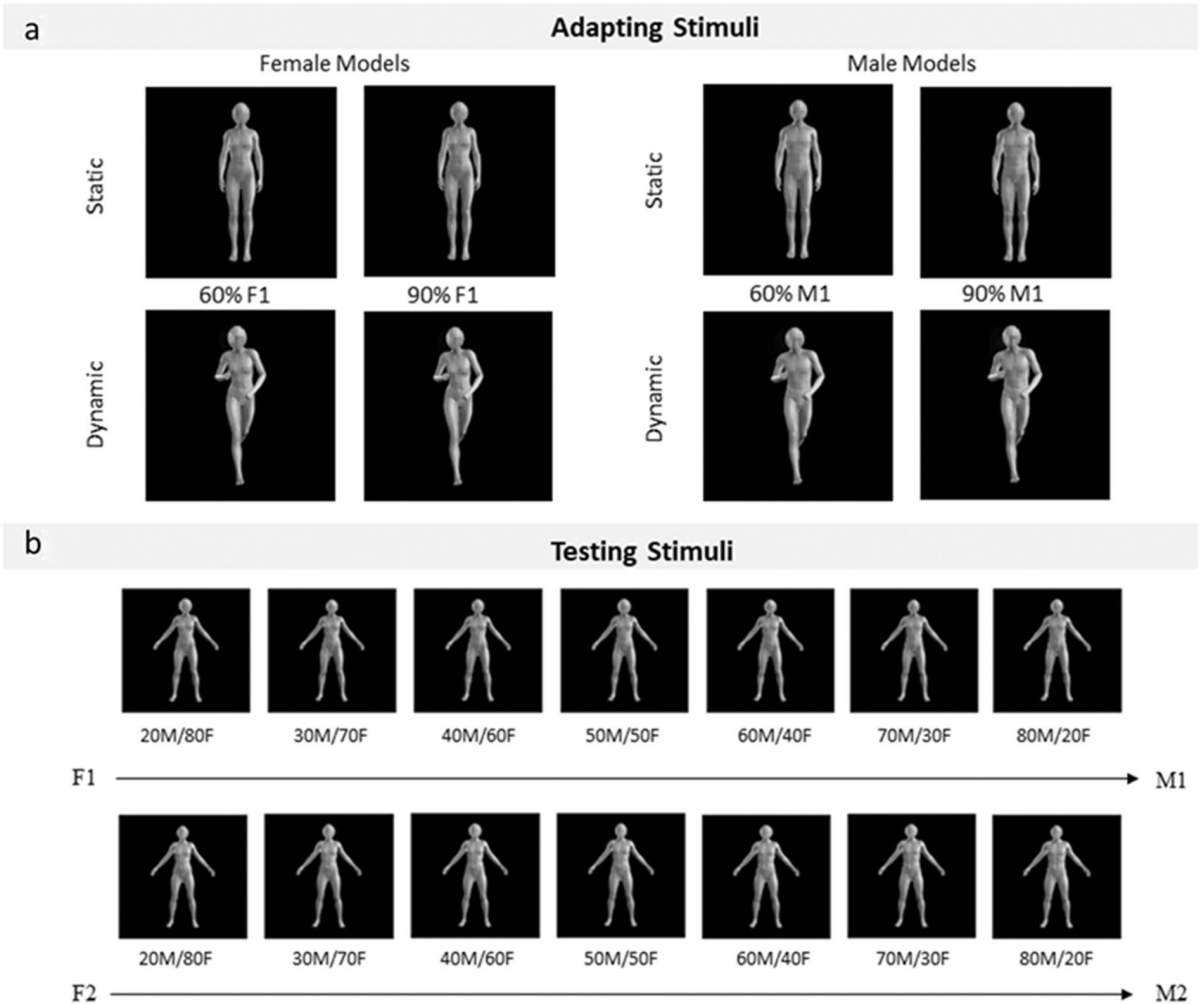
Examples of stimuli used in the study. **a** Examples of female and male virtual models used as Adapting stimuli. The figure depicts the body sex-typing variation (60% vs. 90%) of either static or dynamic female and male models (Identity 1). **b** Androgynous virtual models presented as Testing stimuli. The figure depicts the continuous % variation from 20 male-80 female up to 80 male-20 female for both identities

To generate a set of virtual-human body stimuli systematically controlling for their individual characteristics and masculinity/femininity traits, we used Character Creator 3.0 software (Reallusion, San Francisco, CA, USA). The adapting stimuli were constructed selecting two pairs of male-female virtual-human characters from the software default database (i.e., M_1_–F1 and M_2_–F_2_). Importantly, the male and female character in each pair shared individual bodily characteristics (i.e., those bodily features that make an individual unique independently for gender) but were extremized to along the gender-typing features. Then, we produced two different versions of each character setting the amount of gender traits at 60 or 90%, while leaving neutral the remaining 40 or 10% traits, to obtain more or less masculine/feminine bodies (Fig. 1a). Furthermore, each resulting body stimulus was rendered in ten daily poses, namely five static (e.g., standing, open, idle, and turned postures) and five dynamic poses (e.g., running, walking, jumping, dancing, moving), selected among default postures available in Character Creator. The grey-scale bodies could be viewed from a frontal or three-quarter view and were pictured against a black background. Overall, we had two pairs of male-female characters (M_1_–F1 and M_2_–F_2_) expressing two levels of gender-typicality (60 or 90% of embodied masculinity or femininity traits) rendered in ten postures. Furthermore, images were imported into GIMP 2.10.8 (GNU Image Manipulation Program, Berkeley, CA, USA) to produce a mirrored version of each image. Thus, a total of 80 male and 80 female adapting bodies were obtained. For all images, the head, pectoral and pelvic areas were blurred to mask facial and primary sexual characteristics while keeping enough morphological information to visually convey the sexual phenotype.

**Fig. 2:**
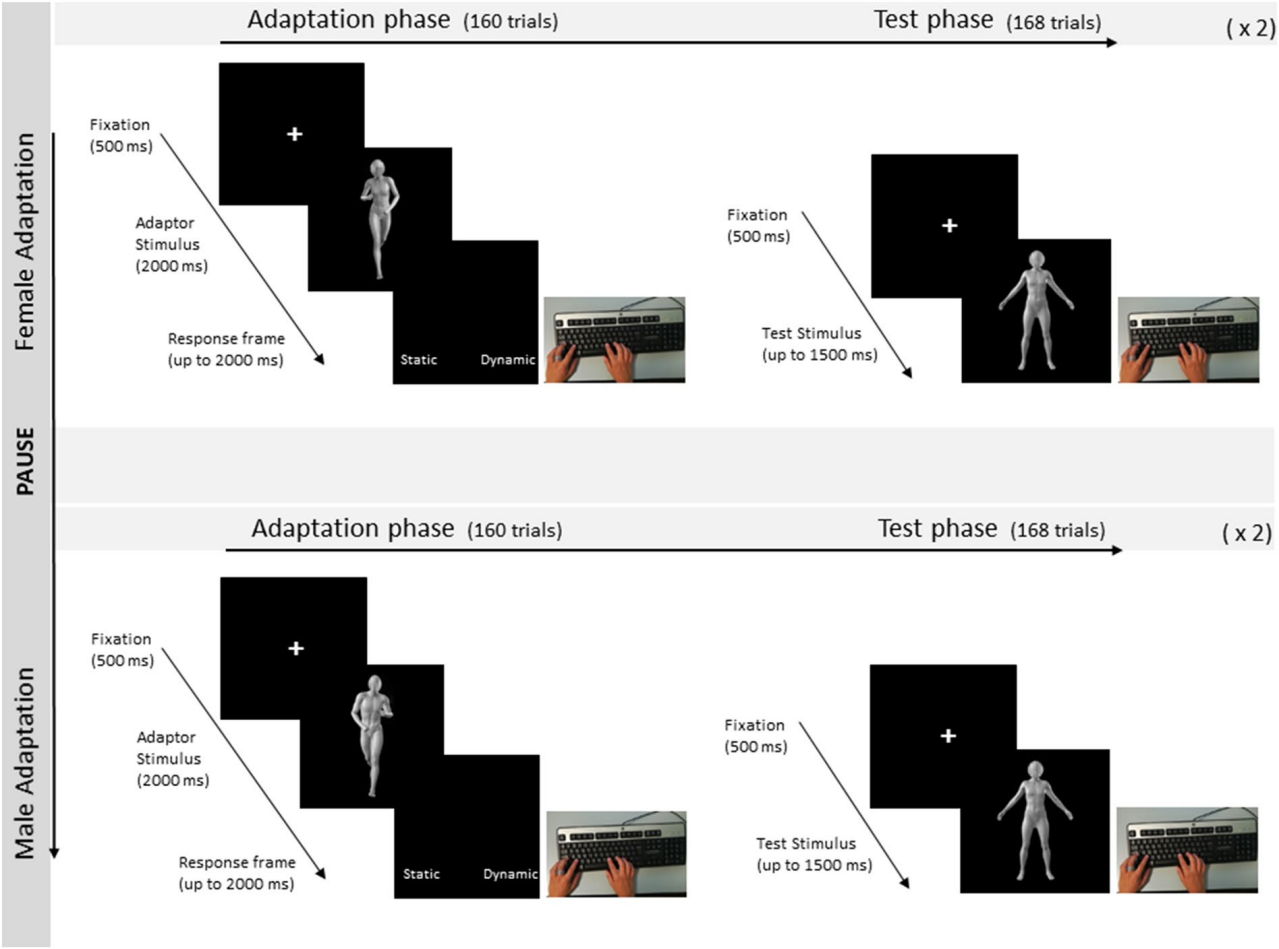
Experimental session and trial procedure. A representation of the timeline of a single Experimental session and a depiction of the events constituting the structure of a trial in the adaptation and test phases. For both Female and Male exposures, two adaptation- and two test-phase blocks were performed

To create the testing stimuli, we again started from the same four characters used for the adapting stimuli. However, this time, to manipulate the percentage of female and male traits expressed by each body, we mixed the two pairs of female and male characters, namely F_1_ with M_1_ and F_2_ with M_2_. The mixing for each pair of characters was applied in a continuous variation from 20% male and 80% female (20M/80F) up to 20% female and 80% male (80M/20F). This led to the creation of androgynous stimuli that, beyond containing different levels of gender-typing features, could contain the individual body characteristics of either the M_1_–F_1_ or the M_2_–F_2_ pair of virtual characters. More specifically, we obtained a total of 7 different percentages of male/female features for each pair of characters (M_1_/F_1_ and M_2_/F_2_; Fig. 1b). All 14 androgynous body figures (2 character pairs × 7 percentages of male/female features) were rendered in the same neutral pose, which was different as compared to those used for the adapting stimuli, and were presented in their original and mirrored versions, for a total of 28 testing bodies.

#### Procedure

The experiment was created with E-Prime software (version 2.0, Psychology Software Tools, Inc., Pittsburgh, PA, USA). Participants sat 60 cm away from a 19-in PC monitor (resolution: 1360 × 768 pixels; refresh frequency: 60 Hz), looking at the 450 × 450 pixels body images presented one at a time at the centre of the screen. Each participant was asked to perform two experimental sessions, one with adaptation to Female models and the other with adaptation to Male models. The order of the two sessions was counterbalanced between participants and a short break was allowed between sessions. Each session was composed of two identical blocks, each one including an adaptation phase and an immediately consecutive test phase. The test phase was identical in all conditions, while the adaptation phase differed across sessions according to whether male or female adapting stimuli were used (Fig. 2).

Each adaptation block comprised 160 trials (i.e., two presentations for each male or female adapting stimulus) and lasted about 8 min. Importantly, one pair of characters (i.e., F_1_/M_1_ or F_2_/M_2_) was presented with a high gender-typicality figure (i.e., 90%), which should lead to higher aftereffects, while the other was presented in a low gender-typicality figure (i.e., 60%), which should lead to lower aftereffects. Each adaptation trial started with the appearance of a white central fixation cross (500 ms) presented on a black background and followed by a body image lasting 2000 ms. Soon after image offset, the Italian words “*Statico*” and “*Dinamico* (*Static and Dynamic* in English) appeared on the bottom of the screen and the participant was asked to report the correct response by pressing the “Z” or “M” key on a QWERTY keyboard. The high- vs. low-adapted identities and the response-key associations were counterbalanced across participants.

Soon after each adaptation block, a test phase block was administered and lasted about 5 min. The 28 possible androgynous bodies were presented six times each for a total of 168 trials per test block; thus, 336 test trials were administered in each experimental session (i.e., 24 trials per cell). In each test trial, a central fixation cross (500 ms) was followed by an androgynous body, which was presented until response or for a maximum of 1500 ms. Participants were asked to report the gender (male or female) of the body stimulus by pressing as soon as possible the key “Z” or “M”. Response-key association was counterbalanced across participants.

#### Data handling

Analyses were performed using Analysis of variance (ANOVA) designs implemented in the STATISTICA software (Stat Soft, Tulsa, OK). The performance in the adaptation phase was not considered in the analysis since it only served to capture participants’ attention to the adapting stimuli. For the test phase, we calculated the proportion of male responses at each level of gender typicality of the androgynous bodies, separately for the male and female adaptation sessions and for the high and low adapting identities. This way, we estimated the increase of male responses according to increasing amount of male/female features. Individual proportions of male responses were fitted as a function of male/female features with a logistic function. Then, for each participant and condition, we calculated the Point of Subjective Equality (PSE), which represents here the level of gender typicality of a stimulus that is equally likely to be judged as a male or a female. Individual PSE were entered into a mixed ANOVA design with Exposure (Male or Female) and Character (High or Low adapting) as within-subject variables and Gender group (Male vs. Female observers) as a between-subjects factor. Significant interactions were explored with Duncan’s post hoc test for multiple comparisons), which has been developed to reduce the risk of false negative (Type II) error when correcting for multiple comparisons (Ijsmi 2016) by reducing the size of the critical difference depending on the number of steps separating the ordered means. This procedure is optimal for testing in the same design effects that may have different sizes (Duncan, 1955; Dunnett, 1980; McHugh, 2011). Significance threshold was set at *p* < 0.05 for all analysis. Effect sizes were estimated with partial eta squared (*η*_*p*_^2^). Values are reported as mean ± standard error of the mean (SEM).

The sample size required for our 2 (Exposure) × 2 (Character) × 2 (Group) mixed within-between ANOVA design was determined with the G*power software (Faul et al. 2009), using the “as in SPSS” option for estimating effect size from *η*_*p*_^2^ and setting the *α* level at 0.05 and the desired power (1 − *β*) at 95%. The expected effect size was set at *f*(U) = 0.71 based on the effect size (*η*_*p*_^2^ = 0.33) of the interaction between exposure and observer’s gender in a previous body-gender-adaptation study (Palumbo et al., 2013).

### Results and discussion

**Fig. 3:**
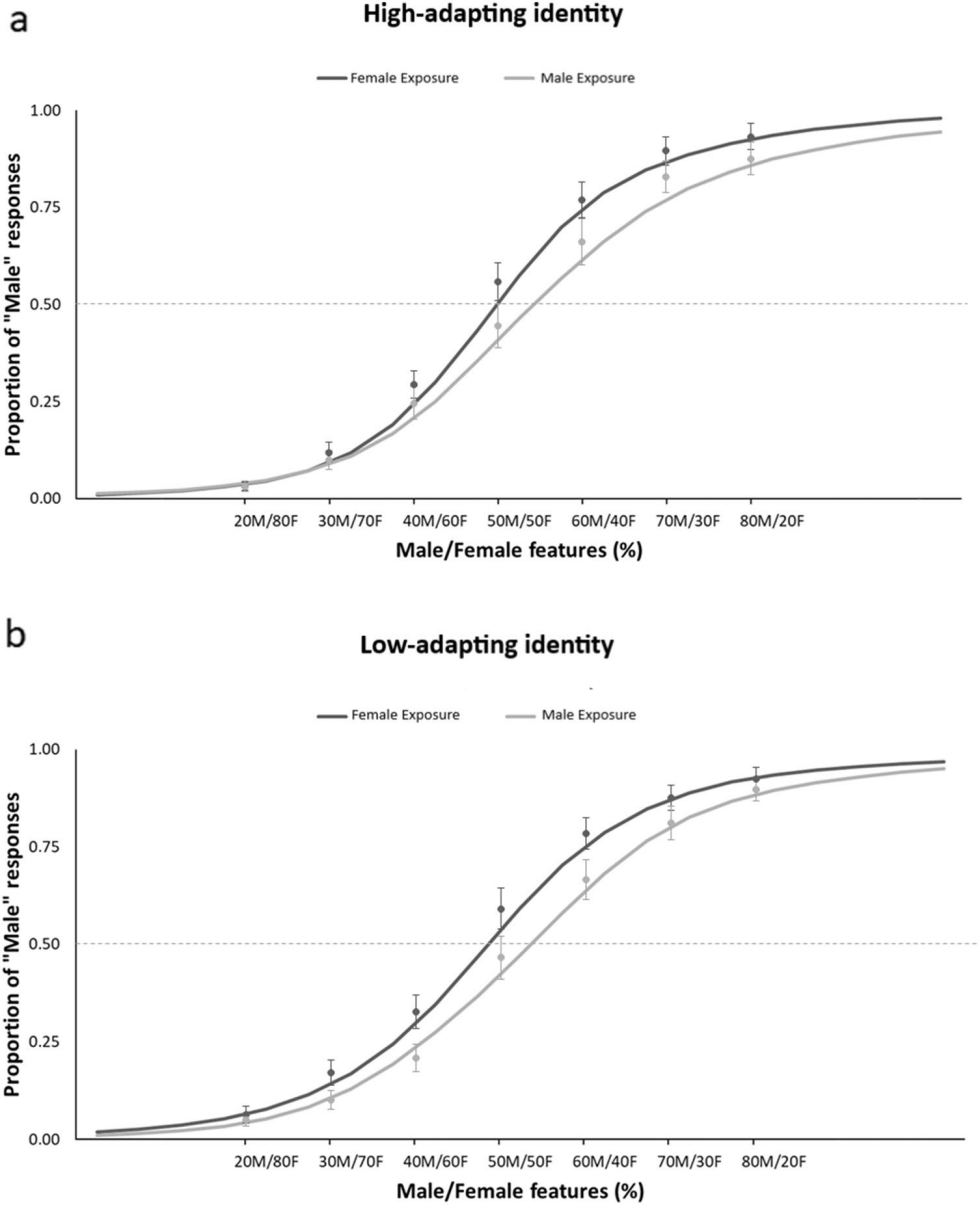
Group data showing the psychometric function of the mean proportion of "male" responses, after adapting to Female or Male bodies, according to increasing % of Male/Female features embodied by the androgynous stimuli used during the test phase in “Experiment 1”. Vertical bars denote standard error of the mean. Results are presented separately for the high-adapting **a** and low-adapted **b** identities

The ANOVA on the PSE scores yielded a significant main effect of Exposure [*F*(1,28) = 6.66; *p* = 0.015; *η*_*p*_^2^ = 0.192], which revealed that, on average, PSE was lower after Female exposure (48.34 ±1.67) than after Male exposure (53.49 ± 2.13). This suggests that participants needed a higher amount of feminine features to judge an androgynous body as female after exposure to feminine bodies than after exposure to masculine bodies. Thus, in keeping with Palumbo and colleagues (2013), our data revealed body-gender aftereffects, in which exposure to a specific gender leads to the judgment of androgynous bodies towards the opposite gender. However, the Character main effect [*F*(1,28) = 1.38; *p* = 0.248; *η*_*p*_^2^= 0.047] and the Exposure × Character interaction [*F*(1,28) < 0.01; *p* = 0.936; *η*_*p*_^2^ < 0.001] were not significant, revealing that participants’ responses were comparably adapted for the High and Low adapting characters (Fig. 3). This is in keeping with previous studies showing the transfer of body size and shape aftereffects across different bodies (Brooks et al., 2016, 2019a; Gould-Fensom et al., 2019; Hummel et al., 2012). Nevertheless, the comparable amount of body-gender aftereffects for both characters contrasts with the findings of a reduction of body size aftereffects when the adapting and testing bodies had different characteristics (Brooks et al., 2016, 2019; Hummel et al., 2012). This reveals that body-gender aftereffects involve populations of neurons that do not code the specific individual characteristics of the observed body. Furthermore, in contrast with the findings of Palumbo et al. (2013), no significant main effect or interaction of the Gender group was obtained (all *F* < 3.24, *p* > 0.08). Thus, participants’ gender was not further considered.

## Experiment 2

Previous studies showed that aftereffects for face distortions (Watson et al. 2003, 2006; Webster & MacLin, 1999) and body size (Brooks et al., 2018) transfer from upright adapting stimuli to inverted testing stimuli. Similarly, body-to-face gender aftereffects also transfer across stimulus orientations (Kessler et al., 2013). In “Experiment 2”, we tested whether body-to-body-gender aftereffects transfer from upright to inverted bodies or whether they are specific for the configural processing of (canonical) upright bodies. To this aim, we adapted participants to female or male upright bodies and tested the aftereffects on the processing of androgynous upright and inverted bodies. If body-gender adaptation selectively involves configural-processing mechanisms, only upright, but not inverted bodies should be adapted. Conversely, if body-gender adaptation affects local processing mechanisms, we expected comparable aftereffects for upright and inverted bodies, since the local processing mechanisms can be used for both upright and inverted bodies. Differently, if both configural and local processing mechanisms are involved, aftereffects should occur for both upright and inverted testing bodies, but they should be lower for the latter.

### Materials and methods

Twenty participants (16 women, mean age 22.2, SD 4.34 years) were recruited. Of them, seven participants participated in Experiment 1 several weeks before. The same stimuli, procedure and data handling approach as in “Experiment 1” were used in this experiment, with the exception that both upright and inverted androgynous bodies were randomly presented in the testing phase. The 28 testing bodies were presented eight times each, four times with an upright orientation and four times with an inverted orientation, for a total of 224 trials per block; thus, 448 test trials were administered in each experimental session (i.e., 16 trials per cell). In addition to the repeated-measure variables of Exposure and Character, here we also tested the effect of the Orientation (upright vs. inverted) of the testing bodies. The sample size required for this 2 (Exposure) × 2 (Character) × 2 (Orientation) repeated-measure ANOVA design was determined by setting the expected effect size was set at *f*(U) = 0.92 based on the effect size (*η*_*p*_^2^ = 0.46) of the interaction between exposure and orientation in the only previous study of the orientation-tuning of body size aftereffects (Brooks et al., 2018).

### Results and discussion

The ANOVA on the PSE values yielded a significant main effect of Orientation [*F*(1,19) = 7.26; *p* = 0.014; *η*_*p*_^2^= 0.28], showing lower PSE for inverted (44.02 ± 3.01) than upright stimuli (52.18 ± 2.35). This suggests that inverted androgynous bodies looked like more masculine since they needed fewer masculine features to be judged as male with respect to their upright counterparts. This unexpected result, however, can reflect a side effect of the exposure procedure, where the adapted upright bodies could be judged as more masculine than the new inverted bodies.

**Fig. 4:**
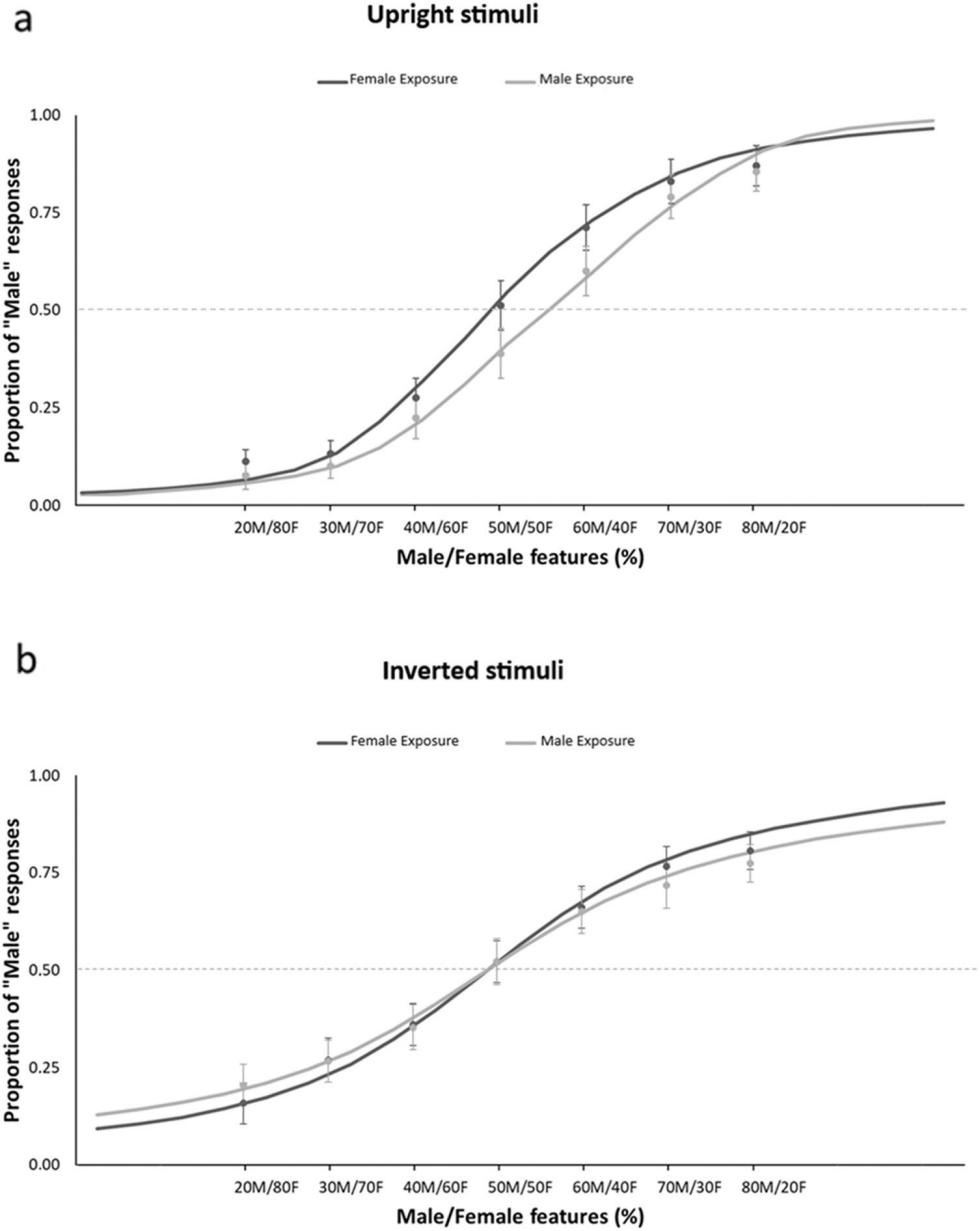
Group data showing the psychometric function of the proportion of "male" responses, after adapting to Female or Male bodies, according to increasing Male/Female features embodied by the androgynous stimuli used during the test phase in “Experiment 2”. Vertical bars denote standard error of the mean. Results are presented separately for upright **a** and inverted **b** testing stimuli

Further, even though the effect of Exposure was not significant [*F*(1,19) = 0.15; *p* = 0.7; *η*_*p*_^2^= 0.008], we found a significant Exposure × Orientation interaction [*F*(1,19) = 6.88; *p* = 0.017; *η*_*p*_^2^= 0.265], which indicated that the effect of adaptation was different for upright and inverted testing bodies (Fig. 4). First of all, Duncan post-hoc comparisons [MSE = 55.092 d*f* = 19] revealed that, in keeping with the main effects of Orientation, PSE scores were significantly lower for inverted than for upright bodies after both Female (*p* = 0.037) and Male (*p* < 0.001) exposures. More importantly, while upright stimuli had lower PSE scores after Female (50.17 ±2.61) than after Male exposure (54.20 ± 2.54; *p* = 0.025), inverted bodies showed no effect of exposure (*p* = 0.21). Thus, in contrast with body size aftereffects (Brooks et al., 2018) and body-to-face gender aftereffects (Kessler et al., 2013), body-to-body-gender aftereffects did not transfer from upright to inverted bodies. On this view, we can address two potential explanations of this finding. On the one hand, body-gender adaptation may involve populations of neurons that are tuned to the specific orientation of the body, thus not being, differently from for faces, completely object-based. On the other hand, it is possible that body-gender adaptation selectively involves configural-processing mechanisms, such that only the processing of upright bodies can be adapted. In keeping with Experiment 1, no main effect or interaction of Character was found (all *p* > 0.434).

## Experiment 3

Here we aimed to qualify the results of Experiment 2 by further investigating the orientation-tuning of body-gender aftereffects. We required participants to recognize the gender of both upright and inverted androgynous models after adaptation to both upright and inverted bodies. Of note, the same type of exposure, namely male or female, was used for the upright and inverted bodies, since we were not looking at evidence of contingent aftereffects across orientations (Rhodes et al., 2004), but at testing the effects of adapting also inverted, and not only upright bodies as in “Experiment 2”. If body-gender adaptation is strictly orientation-tuned, exposure to both upright and inverted bodies is expected to comparably adapt the gender discrimination of upright and inverted androgynous bodies. Conversely, if body-gender adaptation exclusively involves the use of configural processing mechanisms, then we should observe, as in “Experiment 2”, body-gender aftereffects for only upright, but not inverted androgynous bodies even after adapting inverted body perception.

### Materials and methods

Twenty participants (ten women, mean age 23.3, SD 4.78 years) were recruited for this experiment. Of them, two participants also took part in Experiment 1. The same stimuli, procedure and data handling approach as in Experiment 2 were used, but participants were exposed to both upright and inverted distinctively female or male bodies in the adaptation phase. Thus, each of the 80 male or female adapting stimuli was presented once in an upright orientation and once upside-down, for a total of 160 trials.

### Results and discussion

**Fig. 5:**
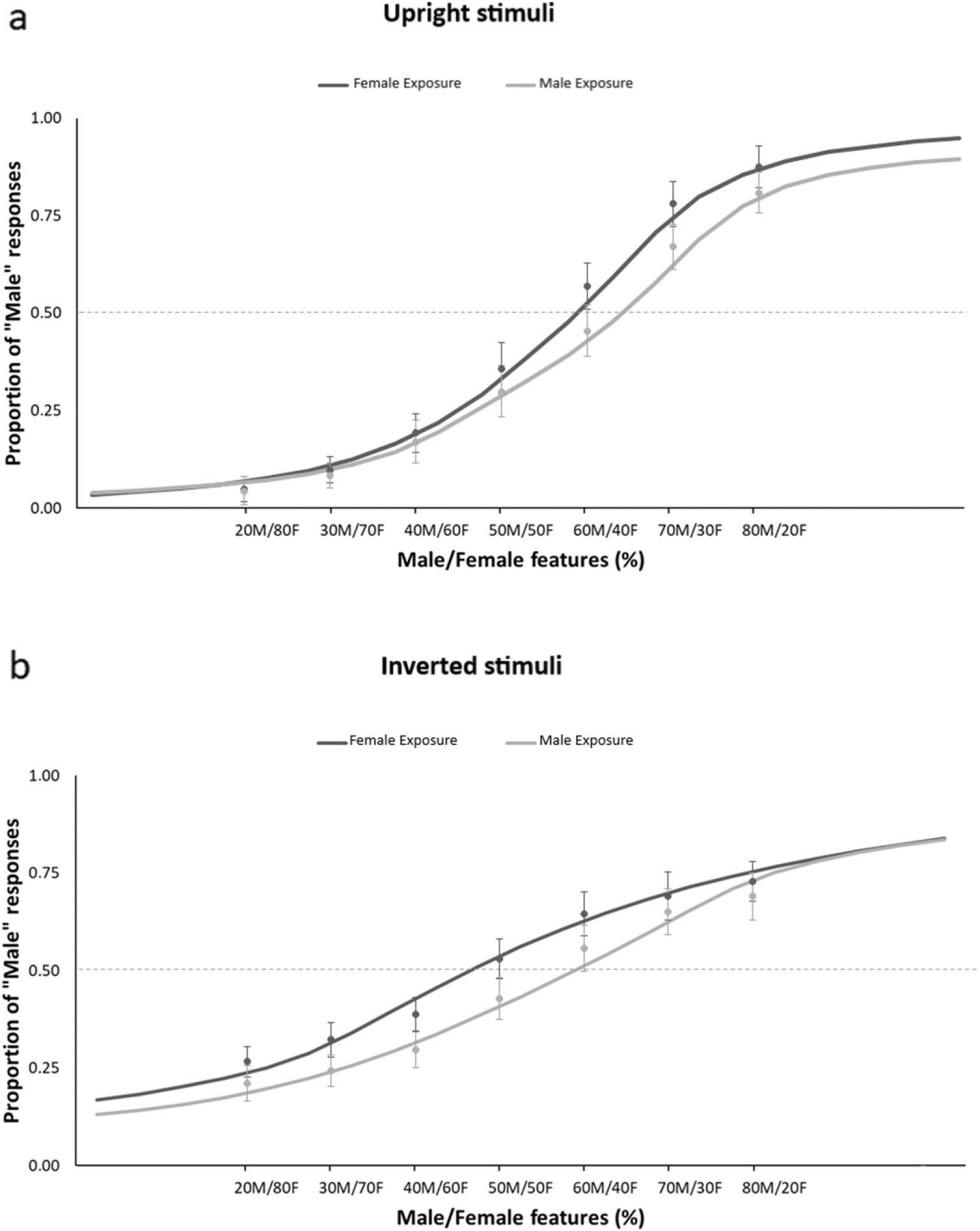
Group data showing the psychometric function of the mean proportion of "male" responses, after adapting to Female or Male bodies, according to increasing male/female features embodied by the androgynous stimuli used during the test phase in “Experiment 3”. Vertical bars denote standard error of the mean. Results are presented separately for upright **a** and inverted **b** testing stimuli

In “Experiment 3”, a significant main effect of Orientation [*F*(1,19) = 7.18; *p* = 0.014; *η*_*p*_^2^= 0.28] was found, highlighting, as in “Experiment 2”, lower PSE for inverted (*M* = 50.68, SEM= 3.11) than upright (56.49 ± 2.33) stimuli. This rules out that the difference between the perceived masculinity of upright and inverted bodies was a side effect of exposure to only upright bodies. Then, a significant main effect of Exposure [*F*(1,19) = 4.94; *p* = 0.038; *η*_*p*_^2^= 0.27] revealed, across orientations, lower PSE after Female exposure (50. 83 ± 2.77) than after Male exposure (56.34 ± 2.85). Crucially and differently from Experiment 2, the interaction between Exposure and Orientation was not significant [*F*(1,19) = 1.73; *p* = 0.203; *η*_*p*_^2^= 0.08]. Thus, exposure to both upright and inverted bodies comparably adapted the gender discrimination of both upright and inverted androgynous bodies, suggesting the orientation-tuning of body-gender aftereffects (Fig. 5). As in Experiments 1 and 2, no main effect or interaction of Character was found (all *F* < 1.36, *p* > 0.2, *η*_*p*_^2^ < 0.002), showing that the same pattern of orientation-dependent adaptation was obtained for both the highly and lowly adapting identities. This points to the identity-independence of the neural populations adapted by both upright and inverted bodies.

## Regression analysis

In Experiments 2 and 3, we also asked participants to fill three questionnaires, in a counterbalanced order at the end of the second experimental session. These questionnaires were the Body Uneasiness Test (BUT), which evaluates body misperceptions (Cuzzolaro et al., 2006), the Autistic Quotient (AQ), which provides a measure of participants’ autistic traits (Baron-Cohen et al., 2001), and the Bem Sex-Role Inventory (BSRI; Bem, 1974), which measures participant’s level of masculinity and femininity. The BUT is a 71-item self-report questionnaire that consists of two parts, one evaluating 6 dimensions related to self-body perception (weight phobia, body image concerns, avoidance, compulsive self-monitoring, detachment and depersonalization) and one looking at specific worries about particular body parts. Here we considered the Positive Symptom Total score (PST), which provides a global measure of a participant’s body satisfaction. The AQ consists of 50 items divided into five subscales measuring either social (i.e., imagination, communication and social skills) or cognitive (i.e., attention to detail and attention switching) aspects of autistic traits (Hoekstra et al., 2008; Warrier et al., 2019). Finally, the BSRI consists of two 20-items subscales measuring masculinity and femininity plus 20 neutral items, aimed at measuring the internalization of different aspects of gender traits.

Using these psychological measures we aimed to test the influence of individual differences in body perception, autistic traits and internalization of gender role on body-gender aftereffects. Indeed, previous studies showed that body size aftereffects are modulated by body misperception in healthy volunteers (Bould et al., 2018; Stephen et al., 2018) and are altered in individuals with eating disorders (Mohr et al., 2016; Mele et al. 2013). Thus, we expected higher aftereffects in individuals with higher levels of body misperceptions. Furthermore, face aftereffects have been shown to be altered in individuals with autism (Pellicano et al., 2007), in particular in those with more severe deficits in the areas of socialization, communication, and restricted and/or repetitive interests (Pellicano et al., 2007), and in healthy individuals with high autistic traits related to social interactions (Fiorentini et al., 2012; Rhodes et al., 2013b). Thus, we expected a diminished sensitivity to adaptation for participants with higher autistic traits, reflecting weaker ability in using prior experience to modulate perceptual experience (Pellicano & Burr, 2012). Finally, even if Experiment 1 showed, in contrast with the findings of Palumbo et al. (2013), no modulation of observer’s gender as a categorical variable, it is still possible that internalized gender role may modulate differential gender body adaptation in men and women. Indeed, it has been widely shown that the degree of masculinity/femininity, rather than biological sex, is a better predictor of body perception and body satisfaction (Cella et al., 2013). Notably, a recent study showed that gender identification, more the biological sex, explains individual differences in autobiographical memory (Compère et al., 2021) and also modulates the initial perceptual processing responses of male and female observers to same vs. opposite gender faces (Domen et al., 2020).

Accordingly, we entered as a predictor into a standard multiple regression analyses two partially independent measures of social autistic traits (i.e., Social skills and Communication; Austin, 2005) and a measure of cognitive autistic traits (i.e., Attention to details), the PST of the BUT, and the Masculinity and Femininity scores of the BSRI. The dependent variable was an individual measure of the magnitude of body-gender aftereffects, calculated as the difference between the PSE after Male exposure and the PSE after Female exposure; the greater the PSE aftereffect index, the greater the repulsion of PSE between the two exposure conditions, the greater the body-gender aftereffects. To obtain a more consistent aftereffect measure across experiments, only participants’ responses for upright stimuli were taken into account, since the adaptation for inverted bodies was different in Experiments 2 and 3.

**Fig. 6:**
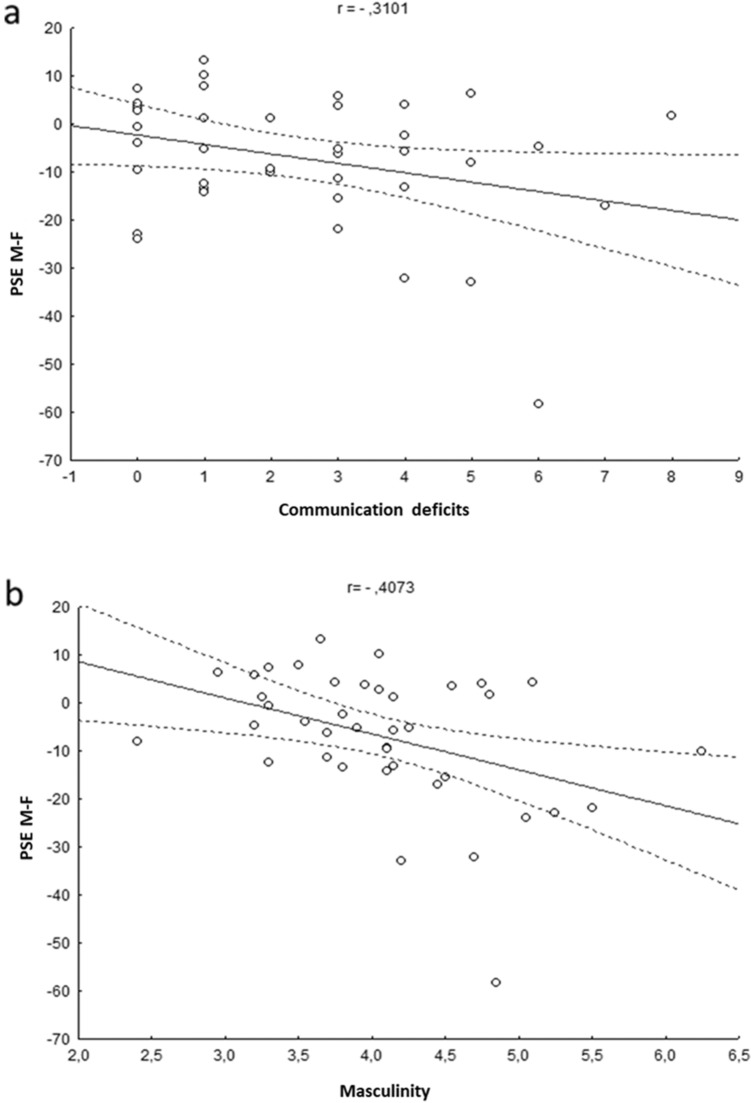
Correlations between the body-gender aftereffect index (calculated as the difference between the Point of Subjective Equality (PSE) after Male exposure and the PSE after Female exposure) and individual scores at the **a** Communication subscale of the Autistic Quotient or **b** Masculinity scale of the Bem Sex Role Inventory

Statistics of standard multiple regression analyses are reported in Table 1. Multicollinearity statistics confirmed that the regression assumption was not violated (Tolerance > 0.65). The whole model was significant (adjusted *R*^2^ = 0.195; *F*(6,33) = 2.57; *p* = 0.037), confirming that the variables considered in the model allowed predicting the amount of individual body-gender aftereffects. In particular, the AQ Communication subscale (*r*(44) = − 0.31; *p* = 0.018) and the Masculinity scale (*r*(61) = − 0.41; *p* = 0.037) were significant predictors, while all the other scale scores were non-significant (all ps > 0.3). Thus, in keeping with the finding of aftereffect alterations for face identity coding in individuals with autism (Pellicano & Burr, 2012) and high autistic traits (Fiorentini et al., 2012; Rhodes et al., 2013b), we had evidence of a modulating role of Communication deficits, which are social aspects of autistic traits (Hoekstra et al., 2008; Warrier et al., 2019), on body-gender adaptation. In particular, greater communication deficits in receiving and providing information (i.e., higher Communication subscale scores), were associated with weaker body-gender aftereffects (Fig. 6A). Furthermore, individuals with higher internalization of a male gender role (i.e., higher Masculinity) also showed weaker body-gender aftereffects (Fig. 6B).

**Table 1:** Multiple Regression results for the body-gender aftereffect index

Coefficients	*β*	*t*	*p* level	Tolerance
**Communication**	− **0.443**	**− 2.493**	**0.018**	**0.654**
Social skills	0.177	1.007	0.321	0.666
Attention to detail	− 0.088	− 0.54	0.593	0.779
Positive Sympthoms Total	0.024	0.142	0.888	0.709
Femininity	− 0.116	− 0.725	0.474	0.81
**Masculinity**	**− 0.38**	**− 2.177**	**0.037**	**0.679**

Bold characters denote significant predictors (*p* < 0.05)

## General discussion

We investigated the identity- and orientation-tuning of the mechanisms involved in body-gender adaptation after exposing participants to prolonged vision of distinctively female or male bodies and then asking them to detect the gender of a series of androgynous bodies. In line with previous researches (Palumbo et al., 2013), we found that exposure to gender-distinctive body models biases the perception of androgynous bodies towards the opposite gender. Differently from the study by Palumbo and co-workers (2013), however, here we collected participants’ judgments on androgynous stimuli after few minutes of exposure to a series of adapting bodies, rather than by alternating, on each trial, adaptor and test stimuli. This allowed us to prove long-lasting adaptation effects after cumulative perceptual experience, thus approaching in a more ecological way the mechanisms of adaptation to body models in everyday life (Brooks et al., 2019).

Further extending previous knowledge, we also showed, across the three experiments, that body-gender aftereffects are independent of the specific individual characteristics of the model, transferring across different body characters. Moreover, we found that adaptation to only upright bodies biased the perception of upright but not of inverted bodies (Experiment 2), while adaptation to both upright and inverted bodies biased the gender perception for both, thus proving the orientation tuning of body-gender aftereffects (Experiment 3). Finally, while in contrast with previous evidence (Palumbo et al., 2013) no effect of the observer’s gender was found, individuals with higher autistic traits and higher internalization of a male gender role displayed less body-gender aftereffects. Conversely, in contrast with our expectations, no effect was found for individual differences in body dissatisfaction. Thus, the findings prompt for the identity independence and orientation tuning of body-gender adaptation mechanisms, which are associated with individual differences in autistic traits and gender identity.

## Body-gender adaptation is identity -independent

Research on visual adaptation has suggested that the gender of faces or bodies is represented mainly by two distinct neuronal channels that are broadly tuned (Ghuman et al., 2010a; Winkler & Rhodes, 2005). In other words, the coding of the two opposite genders follows an entirely symmetrical norm-based system, with male and female faces and bodies represented as opposite poles along the gender dimension and the androgynous body represented as its average value. As for gender, also the representation of body identity follows the lines of norm-based coding (Rhodes et al., 2013a). Indeed, adaptation to a specific body (i.e., “Rose”) biases perception toward the character with opposite features (i.e. “anti-Rose”), thus implementing a sort of opponent coding system. In our experiments, we separately modulated the gender distinctiveness of two different characters, which differed both for their global shape (i.e., height/width ratio and curvature) and for internal details (e.g., skin texture). One character was adapted with a high gender distinctiveness, while the other was adapted with a low gender distinctiveness. Thus, if the neural populations that are adapted by gender exposure code also for individual character features, we expected body-gender aftereffects to be stronger for the highly than for the lowly adapting character. In contrast, in all experiments we found that adaptation to body gender acted regardless of the character. The comparable amount of aftereffects for highly- and lowly- adapting characters suggests that, despite the identity and the gender of the body are susceptible to similar adaptation mechanisms and follow similar norm-based coding, the two body dimensions are processed at different levels of body representations.

This points to a hierarchical organization of body representation, similar to what has been proposed for faces (Bruce & Young, 1986; Haxby et al., 2000; Rhodes et al., 2013a). In this view, body gender is coded preliminarily to other dimensions such as body size and individual characteristics through the adoption of a male or a female body template. In keeping with the view that gender is a low-level dimension on which other relevant cues of the body are grounded, a study on selective-attention demonstrated that whilst gender can be processed independently from body size, the latter cannot be detected independently from the former (Johnstone & Downing, 2017). Accordingly, evidence of contingent body-size aftereffects for male and female bodies (Brooks et al., 2019) suggests that the body size of male and female bodies is coded by separate populations of neurons that are engaged after the access to a male or female template. This hierarchical organization of body processing may also explain the discrepancy between the transfer of body-gender aftereffects across characters and the partial identity- tuning of body-size aftereffects. Indeed, Brooks et al. (2016) investigated the transfer of body-size aftereffects from self- to other- body and showed that, although moderate body size aftereffects occurred across identities, the effects were larger when the adapting and testing stimuli depicted the same person.

Our findings of an identity-independent adaptation of gender perception are in keeping with the response properties of areas in the lateral (extrastriate body area; Downing & Kanwisher, 2001) and medial (fusiform body area; Schwarzlose et al., 2005) occipito-temporal cortex associated with the processing of body form cues (Moro et al., 2008; Peelen & Downing, 2005; Urgesi et al., 2004, 2007). Indeed, fMRI adaptation studies (Kable & Chatterjee, 2006; Wiggett & Downing, 2011) showed that neural activation of these areas during action observation was not adapted with the repetition of the same actor. This suggests that the processing of body form cues in these areas might be used to distinguish individual bodies only at later stages (Downing & Peelen, 2016; Hodzic et al., 2009). Accordingly, our body-gender aftereffects may stem from the adaptation of neural firing in these “low-level” body processing areas, rather than in those involved in-person coding in the anterior temporal cortex (Kriegeskorte et al., 2007; Nestor et al., 2011).

## Body-gender adaptation is orientation-tuned

Results from Experiments 2 and 3 suggested that body-to-body-gender aftereffects are orientation tuned: it was necessary to perceive the adaptor and test stimuli in the same orientation to obtain significant body gender aftereffects. Orientation-independency of aftereffects is held as a marker of the involvement of high-level object-based processing (Brooks et al., 2018; Watson & Clifford, 2006). Thus, our finding of an orientation tuning of body-gender aftereffects speaks against the involvement of fully object-based mechanisms in body-gender adaptation. This is in contrast with the findings of a transfer across orientations of face-to-face (Watson et al. 2003, 2006; Webster & MacLin, 1999) and body-to body (Brooks et al., 2018) shape aftereffects. More importantly, a previous study (Kessler et al., 2013) also documented an orientation transfer of body-to-face gender aftereffects, wherein either upright or inverted bodies adapted the gender perception of both upright and inverted faces. This last finding suggests that gender cues conveyed by the body activate orientation invariant representations of face gender. Conversely, in our study, we found that perception of body gender activates orientation-tuned representations of body gender. The discrepancy between the body-specific and cross-categorical aftereffects may point to the involvement of different levels of gender coding, one more conceptual and independent from the specific person cues, either facial or bodily (Gaetano et al., 2014), and another more perceptual and specifically tuned to the specific stimulus template (Kovács et al., 2006). This is in keeping with the evidence of contingent face-to-face gender aftereffects for upright and inverted faces (Rhodes et al., 2004), which may reflect selective adaptation of configural and local processing mechanisms of face perception. In a consistent way, our results suggest that body-gender adaptation selectively engages neural populations that are tuned to the processing of upright or inverted bodies and are involved in configural and local processing of bodies, respectively.

Notably, Brooks and co-workers (2018) found that a significant, still weakened, transfer of body-size and -shape aftereffects can be obtained when the adaptor and test stimuli are tilted 90° away. This suggested that the mechanisms mediating body shape and size aftereffects operate within an object-centered frame of reference. However, the body stimuli used in this previous study were tilted at a +45° or − 45° angle, a manipulation that, while reducing retinotopic contributions, cannot disentangle the engagement of configural and local processing. It is indeed likely that ±45°-tilted bodies involve a mix of configural and local processing. Furthermore, as mentioned above, it is possible that body gender and body size are encoded at different levels of body representations, as demonstrated by opposite body-size aftereffects contingent on the gender of the stimuli (Brooks et al., 2020). On this view, our findings are again in line with a hierarchical organization between body gender and size coding, wherein gender processing is driven by low-level, identity-independent, and orientation-tuned mechanisms, while body size processing involves high-level, object-based mechanisms, which are, at least partially, tuned to the individual characteristics of the body (Brooks et al., 2016) and independent from its orientation.

## Body-gender adaptation is associated with masculinity and autistic traits

A previous study reported that, despite both men and women were adapted by models of either gender, aftereffects were stronger after adaptation to a model of the same gender (Palumbo et al., 2013). This was explained as the enhancement of perceptual attributes of the other gender after a prolonged vision of one’s own gender and was qualified as an attentive advantage for mate selection. However, we did not find any differences between men and women. This contrasting finding might relate to the type of gender features adapted in the two studies. Indeed, Palumbo et al. (2013) used body silhouettes and only manipulated the distinctiveness of the global male and female figures (i.e., shoulder, torso, and hip width). In contrast, we used 3-D body renderings and, even if primary sexually-dimorphic cues (i.e., breast and genitals) were blurred, we manipulated not only the global body figure (i.e., width of shoulder, torso and hip and their relationship, such as the waist-to-hip ratio), but also the internal secondary sexually-dimorphic cues (i.e., musculature, body fatness), which also characterize gender perception from faces (Skomina et al., 2020). Indeed, such a gender-contingent perceptual asymmetry has not emerged in the case of face gender adaptation, despite of the fact that faces appear to be more relevant than bodies for the evaluation of others’ attractiveness (Currie & Little, 2009).

Nevertheless, we found a significant effect of the individual internalization of a male gender role on the amount of body-gender aftereffects, with individuals with higher self-reported masculinity showing weaker aftereffects. The effects of levels of masculinity/femininity on prevalence of body dissatisfaction and ED behaviors have been previously reported (Johnson et al., 1996; Murnen & Smolak, 1997; Sitnick & Katz, 1984). In particular, a negative correlation between masculinity and EDs has been documented, independently from biological gender (Cella et al., 2013), thus evidencing masculinity as a protective factor against the development of EDs in both men and women. Our finding of a negative association between masculinity and body gender aftereffects may shed a new light on how masculinity may protect from body dissatisfaction and EDs. Indeed, stronger male-gender internalization may be associated with a weaker reshaping of body perception, lower influence of body ideals and, consequently, less body misperception. Even if we did not find a reliable association between the amount of body-gender aftereffects and body dissatisfaction, as measured with the BUT, it is possible that other aspects of ED traits and symptoms may be more strongly associated with atypical body adaptation (Mele et al., 2016; Cazzato et al. 2016).

In this regard, we found that higher autistic traits related to communication deficits, but not those related to attention to details, predicted weaker body-gender aftereffects. On one hand, this result is in keeping with the findings of reduced face aftereffects in individuals with autism (Pellicano et al., 2007) and in their relatives (Fiorentini et al., 2012) as well as in individuals with higher autistic traits (Rhodes et al., 2013b). In particular, Rhodes and colleagues (2013b) found that the amount of face-identity aftereffects was negatively associated with the social aspects of autistic traits in male, but not female undergraduate students. In keeping with our findings, no effect was instead documented for the attention-to-detail scale. Thus, our results extend these aforementioned studies by showing that alterations of not only face but also body adaptation mechanisms may be an endophenotype for autism. On the other hand, the reduced body adaptation in individuals with higher autistic traits may also inform on the aspects of EDs that may be associated with altered body adaptation mechanisms. Indeed, the increased interest on the neurocognitive profile of individuals with EDs has shed light on a conceivable association between autistic traits and anorexia nervosa symptoms (Roberts et al., 2011; Tchanturia et al., 2013). On this perspective, our result is in line with the hypothesis that deficits in social communication, which are shared by patients with EDs and with autism, may influence the perceptual phenomenon of visual adaptation and body perception. Future studies, however, are needed to substantiate the alterations of body gender adaptation in clinical populations with EDs or autism.

## Inverted bodies are perceived as more masculine

Unexpectedly, we found that independently from adaptation, inverted bodies were judged as more masculine than upright bodies. This result is in contrast with the finding for face gender discrimination (Rhodes et al., 2004), where inverted faces were judged as more feminine that upright faces. It may appear also in contrast with the sexualized-body inversion hypothesis, which claims a reduced use of configural processing for female with respect to male bodies, as revealed by lower inversion effects for female than male models (Bernard et al., 2012, 2015). Indeed, inverted bodies should appear more feminine, while we found the opposite. However, this effect has been attributed to an object-like processing of female bodies portrayed in sexualized clothes and poses, as typical inversion effects have been observed for personalized representation of either men and women (Cogoni et al., 2018), and may not apply to our virtual-human models presented in neutral postures. Rather, the fact that we found a tendency to report a male body for inverted stimuli may reflect the emergence of a general male decision bias that occurs when gender decision is particularly difficult, as in the case of inverted bodies. This bias has been demonstrated for several gender cues, including faces (Wild et al., 2000), body shapes (Gandolfo & Downing, 2020; Johnson et al., 2012) and biological motion displays (Troje & Szabo, 2010).

## Limitations and future directions

Limitations of our study need to be considered. First of all, we did not test participants’ judgment of androgynous bodies without previous exposure, but we tested only the difference between the two exposure conditions. This was aimed at reducing the repetition of judgments within the same participants but did not allow us to exclude preexisting decision biases in reporting the presence of male and female bodies. In fact, the difference between inverted and upright bodies, independently from exposure, might point to these biases. Secondly, the results could be limited to the specific type of stimuli used, namely human-like avatars, and a different pattern of results may be driven by the use of silhouettes and photographs of real person bodies. Then, a relatively long exposure time during the test phase (i.e., 1500 ms) was used, but we did not control for the strategies adopted by participants during visual inspection (e.g., Cogoni et al., 2018). Finally, although we compared the effects of adaptation to only upright or to both upright and inverted bodies, we did not test the effect of exposure to only inverted bodies. Thus, we could not isolate the effects of adapting only feature-based mechanisms on body gender perception. Future studies are needed to further elucidate the characteristics of body-gender adaptation and to deepen our knowledge on how we extract information on the gender of conspecifics based on bodily cues.

Nevertheless, our study qualifies the description of body gender perception, highlighting that its experience-dependent reshaping is based on mechanisms that are independent from the specific person’s characteristics but tuned to the use of configural or local processing. Furthermore, they also provide hints to the understanding of how the alterations of the malleability of body perception may be associated with individual difficulties in social interactions.

## Data Availability

The datasets generated and analysed during the current study are available from the corresponding author on reasonable request.

## Code availability

The E-prime code used during the current study is available from the corresponding author on reasonable request.
